# Biopsy and Tracheobronchial Aspirates as Additional Tools for the Diagnosis of Bovine Tuberculosis in Living European Bison (*Bison bonasus*)

**DOI:** 10.3390/ani10112017

**Published:** 2020-11-02

**Authors:** Anna Didkowska, Blanka Orłowska, Lucjan Witkowski, Katarzyna Olbrych, Sylwia Brzezińska, Ewa Augustynowicz-Kopeć, Monika Krajewska-Wędzina, Andrzej Bereznowski, Wojciech Bielecki, Michał Krzysiak, Alicja Rakowska, Wanda Olech, Michele A. Miller, Wade R. Waters, Konstantin P. Lyashchenko, Krzysztof Anusz

**Affiliations:** 1 Department of Food Hygiene and Public Health Protection, Institute of Veterinary Medicine, Warsaw University of Life Sciences (SGGW), Nowoursynowska 159, 02-776 Warsaw, Poland; blanka_orlowska@sggw.edu.pl (B.O.); krzysztof_anusz@sggw.edu.pl (K.A.); 2 Laboratory of Veterinary Epidemiology and Economic, Institute of Veterinary Medicine, Warsaw University of Life Sciences (SGGW), Nowoursynowska 159, 02-776 Warsaw, Poland; lucjan_witkowski@sggw.edu.pl (L.W.); andrzej_bereznowski@sggw.edu.pl (A.B.); alicja_rakowska@sggw.edu.pl (A.R.); 3 Department of Morphological Sciences, Institute of Veterinary Medicine, Warsaw University of Life Sciences (SGGW), Nowoursynowska 159, 02-776 Warsaw, Poland; katarzyna_olbrych@sggw.edu.pl; 4 National Tuberculosis Reference Laboratory, Department of Microbiology, National Tuberculosis and Lung Diseases Research Institute, Płocka 26, 01-138 Warsaw, Poland; s-brzezinska@wp.pl (S.B.); e.kopec@igichp.edu.pl (E.A.-K.); 5 Department of Microbiology, National Veterinary Research Institute, Partyzantów 57, 24-100 Puławy, Poland; kappa2@wp.pl; 6 Department of Pathology and Veterinary Diagnostics, Institute of Veterinary Medicine, Warsaw University of Life Sciences (SGGW), Nowoursynowska 166, 02-787 Warsaw, Poland; wojciech_bielecki@sggw.edu.pl; 7 Institute of Forest Sciences, Faculty of Civil Engineering and Environmental Sciences, Bialystok University of Technology, Wiejska 45 E, 15-351 Białystok, Poland; morbital@interia.eu; 8 Białowieża National Park, Park Pałacowy 11, 17-230 Białowieża, Poland; 9 Department of Animal Genetics and Conservation, Institute of Animal Sciences, Warsaw University of Life Sciences (SGGW), Ciszewskiego 8, 02-786 Warsaw, Poland; wanda_olech@sggw.edu.pl; 10 Department of Science and Innovation-National Research Foundation Centre of Excellence for Biomedical TB Research, South African Medical Research Council Centre for Tuberculosis Research, Division of Molecular Biology and Human Genetics, Faculty of Medicine and Health Sciences, Stellenbosch University, P.O. Box 241, Cape Town 8000, South Africa; miller@sun.ac.za; 11 National Animal Disease Center, Agricultural Research Service, United States Department of Agriculture, 1920 Dayton Avenue, Ames, IA 50010, USA; randtwaters@hotmail.com; 12 Chembio Diagnostic Systems, Inc., 3661 Horseblock Road, Medford, NY 11763, USA; kLyashchenko@chembio.com

**Keywords:** biopsy, European bison, *Mycobacterium caprae*, tracheobronchial aspirates, tuberculosis

## Abstract

**Simple Summary:**

In this study, additional methods of collecting material for bovine tuberculosis diagnosis in living European bison were introduced. We showed a potential usage of tracheobronchial aspirates and ultrasound-guided biopsies from lateral retropharyngeal lymph nodes in living animals for diagnostics. We confirmed that the isolation of *Mycobacterium caprae* in living European bison is possible, as is the respiratory shedding of viable *M. caprae* in this host. This study is important as tuberculosis is a real threat for European bison which is an endangered species and the improvement of diagnostics can help with better health monitoring and further restitution.

**Abstract:**

The diagnosis of bovine tuberculosis (BTB) in living wildlife remains a complex problem, and one of particular importance in endangered species like European bison (*Bison bonasus*). To identify infection and avoid the unnecessary culling of such valuable individuals, current best practice requires the collection and culture of material from living animals, as mycobacteria isolation remains the gold standard in BTB diagnosis. However, such isolation is challenging due to the need for the immobilization and collection of appropriate clinical material, and because of the sporadic shedding of mycobacteria. In the present study, we evaluated the potential of sampling for the detection of BTB in a group of seven living European bison suspected of being infected with *Mycobacterium caprae*. The specimens were collected both as swabs from the nasal and pharyngeal cavities, tracheobronchial aspirates (TBA), ultrasound-guided biopsies from lateral retropharyngeal lymph nodes, and post mortem, from mandibular, retropharyngeal and mediastinal lymph nodes. Clinical samples were tested for mycobacterial species via mycobacteriological culture and PCR. *M. caprae* was isolated from collected material in two out of four living infected individuals (TBA, biopsy) and mycobacterial DNA was detected in three out of four (TBA, pharyngeal swab) bison. This is the first report of isolation of *M. caprae* in living European bison. Our findings demonstrate the value of diagnostic tests based on both molecular testing and culture in European bison and confirm the respiratory shedding of viable *M. caprae* in this host species.

## 1. Introduction

Bovine tuberculosis (BTB), caused primarily by *Mycobacterium bovis* or *Mycobacterium caprae* presents a significant threat not only to livestock, but also to wildlife. Although post mortem tests are the basis for monitoring the health of wildlife [1], the importance of diagnostic tests in living animals is increasing [2]. In Poland, there is a clear need to develop diagnostic methods for the detection of BTB in living European bison (*Bison bonasus*, EB) which were near extinction 90 years ago and have since been reintroduced to their natural habitat. The successful restitution of this species presents a number of complex challenges, such as its limited gene pool, the need to set up and locate new herds, a number of environmental hazards, and their susceptibility to numerous infectious diseases including BTB [3,4,5,6]. 

Hence, there is a need to develop diagnostic methods for BTB, particularly given the recent increase in the number of cases observed in EB in Poland [7,8,9,10] and the need to protect valuable individuals from unnecessary culling. Traditionally, assays based on cell-mediated immune responses, such as gamma-interferon [11] and tuberculin skin tests, are used for the diagnosis of BTB in livestock but present logistical difficulties when used in wildlife, such as the requirement for capture, multiple immobilizations for performing the tuberculin skin test, and the lack of validated tests, leading to issues with interpretation of results. For BTB diagnostics in wildlife, one of the main challenges in using diagnostic tests based on immunological methods remains the identification of specific biomarkers for specific species [12,13]. Therefore, many existing diagnostic methods for living wildlife, including EB, are based on serological tests [14,15]. However, with tests based on immunological responses, there is a risk of getting false positive results, e.g., due to cross-reactions with non-tuberculous Mycobacteria [16] or false negative results, e.g., as a result of infections with immunodeficiency viruses or initial phase of infection [17,18]. High specificity of tests is extremely important in endangered species and even though not noted often, there have been cases of culling animals positive in host-based immunological tests which appear negative in post mortem culture (Didkowska, unpublished data).

Due to above limitations, there is a need to develop advanced techniques to improve the diagnosis for infectious diseases in captive wildlife kept in zoos, rehabilitation centers and private farms, especially when working with valuable and unique individuals under legal protection (e.g., EB kept in closed-breeding centers). The most reliable methods of confirming that an animal is BTB-positive are those that detect infection directly, such as mycobacterial isolation. In human medicine, acid-fast stain microscopy, mycobacterial culture and PCR analysis are frequently used to directly confirm the presence of a *Mycobacterium tuberculosis* complex (MTBC), and these methods play a crucial role in tuberculosis diagnosis. In these cases, the clinical material is primarily sputum collected over the course of three days [19]. In veterinary medicine, such direct methods are mainly used as part of post mortem diagnosis [20,21], as sampling in live animals is difficult [1]. Nevertheless, in some species, attempts have been made to perform a culture or PCR analysis of clinical samples collected in living animals [22,23]. Therefore, the aim of this study was to isolate mycobacteria from material collected from living EB suspected of being infected with *M. caprae*, and identify the isolates based on their genetic material.

## 2. Materials and Methods

### 2.1. Material Collection 

Clinical material was collected from seven EB with suspected *M*. *caprae* infection. Animals (5 females, 2 males) came from two ex situ breeding centers, their age ranging from 5 to 19 years. Each individual was subjected to pharmacological immobilization as previously described [24] to collect tracheobronchial aspirates (TBA), nasal and pharyngeal swabs, and biopsies of the lateral retropharyngeal lymph nodes. Samples from the nasal and pharyngeal cavity were collected with sterile swabs (Equimed, Kraków, Poland). These were transported to the laboratory on AMIES transport media (Equimed, Kraków, Poland) or moistened with physiological solution media (Polfa Lublin, Lublin, Poland).

TBA were collected by nasotracheal intubation. After straightening the neck in the sagittal plane (neck and head in line) a silicone gastric tube type F21/1200 catheter (TOMEL-TD, Tomaszów Mazowiecki, Poland) was introduced through the nostril. The catheter measured 120 cm in length and 7 mm in diameter, with side and central holes. After introducing the catheter, 200 mL of sterile physiological saline solution (Polfa Lublin) was administered in the area of the tracheal bifurcation. The fluid was immediately aspirated using a 100 mL syringe. The aspirate obtained, with a volume of 10–30 mL, was stored in sterile tubes at 4 °C and transported to the laboratory.

**Figure 1:**
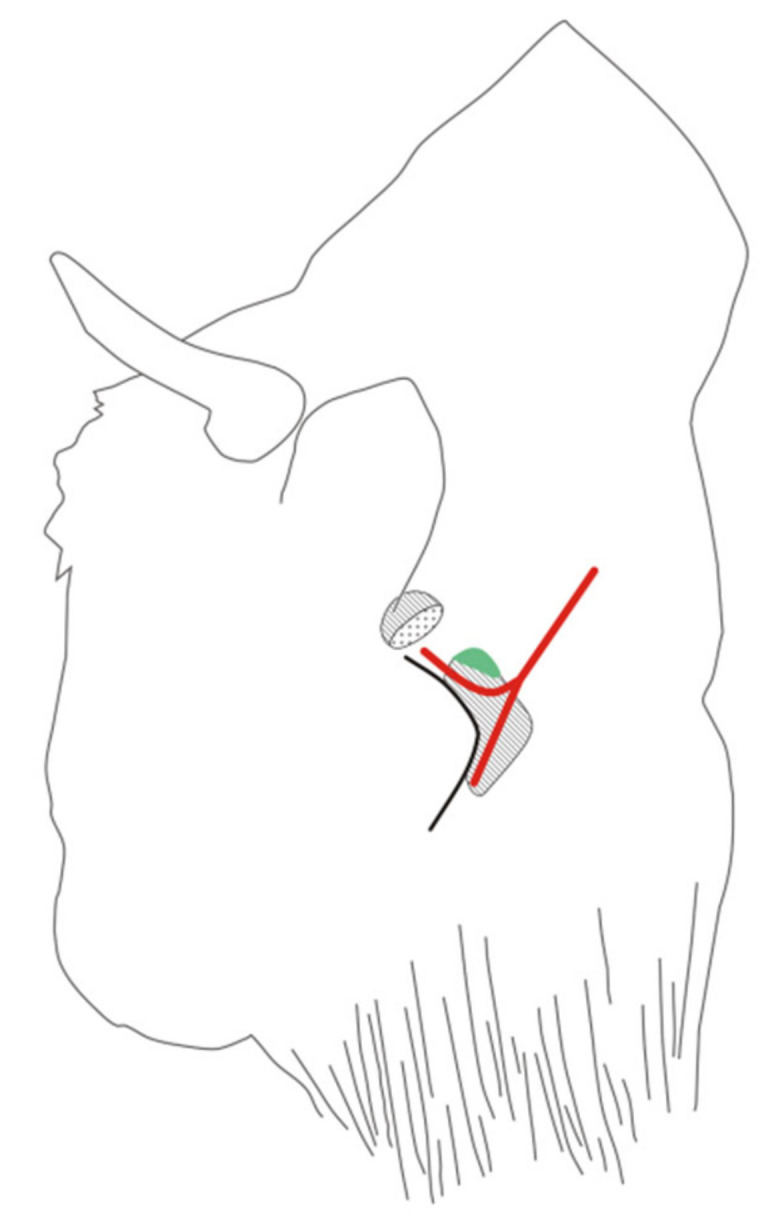
The location of the biopsy needle insertion in the European bison, performed to collect a fragment of the retropharyngeal lateral lymph node. Legend: hatched organs: above the projection of the parotid gland, below the projection of the mandibular gland. The projection of the lateral retropharyngeal lymph node is given in green. Red lines: upper—projection of the maxillary vein, lower—projection of the lingual–facial vein. The maxillary vein and facial–linguistic vein converge into the external jugular vein.

In order to collect a fragment of the lateral retropharyngeal lymph node, a thick-needle biopsy was performed. For material collection, the EB was placed in lateral recumbency with the neck extended. The procedure was started by palpating the lateral retropharyngeal lymph node under the caudal margin of the mandibular gland. The location was confirmed using a Dramiński 4VET slim ultrasound system with a 60 mm and 7.5 MHz abdominal linear head (Dramiński, Olsztyn, Poland). The puncture site was located behind the mandible, between the atlas wing and the maxillary vein, slightly above 1/2 of the length of the mandible branch (Figure 1). 

**Figure 2:**
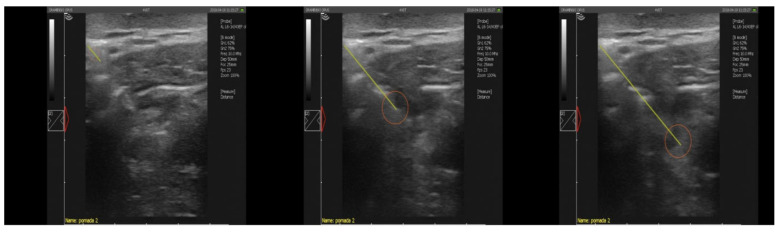
Ultrasound image of the insertion of the biopsy needle into the lateral retropharyngeal node in the European bison (Dramiński 4VET slim with 60 mm and 7.5 MHz abdominal linear head). Legend: yellow line—the course of the needle puncture, red circle—place of material collection.

Before performing the punctures, hair was shaved and skin disinfected with alcohol. A Quick-Core Biopsy Set (Cook Medical, Bloomington, IN, USA) biopsy needle with a 15 cm coaxial kit was used to collect the sample. The head of the ultrasound was applied at 1/3 of the length of the mandible branch, below the wing of the apical vertebra, in the direction of the head. The course of the puncture was monitored by ultrasound by inserting the coaxial set, which was the guide for the biopsy needle (Figure 2). The puncture was performed at a depth of 5–7 cm. The needle was directed toward the head under the mandibular branch of the lingual –facial vein. The tip was gently inserted into the lymph node after overcoming the resistance of its capsule. In order to collect as much material as possible, the needle was rotated around its long axis before releasing the plunger. The piston was then released and the needle removed. The collected material was suspended in 1 mL of sterile physiological solution (Polfa Lublin, Poland) and transported in sterile tubes to the laboratory at 4 °C.

After material collection, animals were immediately euthanized due to previous studies indicating MTBC infection or due to being suspected of infection due to contact with TB-positive animals. All procedures were conducted in accordance with Polish applicable regulations. Euthanasia was conducted according to the Decision of The Polish Ministry of The Environment number DOP-WPN.286.219.2018.MŚ (6 animals). For one animal (from zoo) decision was not necessary according to Polish law. Ante-mortem collection of material does not need approval decision due to Polish law and was conducted by qualified veterinarians maintaining animals welfare. During post mortem examination, mandibular, retropharyngeal and mediastinal lymph nodes from each EB were collected and transported at 4 °C to the laboratory. 

### 2.2. Culture

The methodology used for mycobacterial culture was consistent with that commonly used in tuberculosis reference laboratories [25]. Samples obtained from TBA and swabs were centrifuged (1500 g, 10 min). The tissue materials (biopsy and lymph nodes collected post mortem) were minced using sterile scissors. Then, the tissues were transferred to a glass mortar and ground with physiological saline. The materials (crushed lymph nodes and biopsies, swabs) were decontaminated using NaCl–NaOH. 

In each case, the resulting pellet was plated on two solid media: Stonebrink (Becton Dickinson, Franklin Lakes, NJ, USA) and Löwenstein-Jensen (Becton Dickinson). The inoculated plates were incubated at 37 °C. Growth was assessed every seven days for 12 weeks. If rough white to light yellow colonies were noticed, they were considered as *Mycobacterium* sp. 

After decontamination, the material from the resuspended pellet was also inoculated into liquid Middlebrook7H9 medium, consisting of a BBL MGIT mycobacterium growth test tube enriched with growth additive (Becton Dickinson) [25]. The tubes were placed in a BACTEC™ MGIT™ 960 Mycobacterial Detection System (Becton Dickinson) and incubated at 37 °C. Fluorescence readings were taken continuously by a UV transilluminator for six weeks. 

### 2.3. ProbeTec 

Detection of MTBC DNA directly from the clinical material was performed using the BD ProbeTec *Mycobacterium tuberculosis* Complex (DTB) Direct Detection Reagent Pack (Becton Dickinson) according to the manufacturer’s instructions. For use with tissue materials, an additional step was introduced; genetic material was purified using the automatic Maxwell^®^ Clinical Sample Concentrator (CSC) Instrument (Promega Corporation, WI, USA) according to the manufacturer’s instruction. Therefore, for tissue materials, the ProbeTec test thermal inactivation step was omitted. 

### 2.4. DNA Isolation and Molecular Identification 

Material from liquid media was centrifuged before further analysis (13,000 rpm, 10 min). Material from solid media was used directly. DNA isolation and molecular identification of strains were performed as described previously [26]. Briefly, DNA was isolated using a Genolyse isolation kit (Hain Lifescience, Nehren, Germany). Strains were differentiated using the GenoType^®^MTBC assay (Hain Lifescience) and subjected to spoligotyping (Genutaur molecular products, Kampenhout, Belgium). All tests were carried out in accordance with the manufacturer’s instructions. 

## 3. Results

### 3.1. Culture

From the samples, *M. caprae* was isolated from two out of the seven living EB; two isolates were obtained from biopsies and one from TBA. More infected animals were identified by the post mortem samples with *M. caprae* isolated from four out of seven EB (Table 1). The results obtained on solid media confirmed those on the liquid media. 

### 3.2. Probetec

Using the samples collected from living animals, MTBC genetic material was detected in two out of seven EB; one being positive on the TBA and pharyngeal swab, and the other, only on TBA. The PCR detected MTBC genetic material in four of seven EB using post mortem samples (Table 1). 

### 3.3. Molecular Identification 

Speciation of all isolated strains identified *M. caprae*–spoligotype M. bovis _4_ CA 1600 (octagonal pattern: 200003770003600) (SpolDB4 database).

## 4. Discussion

Although culture test material is rarely collected from living wildlife, this method is considered the gold standard for tuberculosis diagnosis, and attempts should be made to isolate MTBC bacteria from clinical samples [27]. However, this approach has some disadvantages, of which the most troublesome is the long waiting time (6–12 weeks) for results [28]. In addition, obtaining the clinical material can be difficult, and the location of tuberculous lesions varies between species and individuals, which may lead to false negative results. Furthermore, it is challenging to collect material from wildlife repeatedly over several days, which can also reduce the sensitivity of analyses due to intermittent mycobacteria shedding [29]. Finally, to obtain the most conclusive results, sampling for mycobacterial culture should be performed from as many potentially affected tissues/organs as possible [30]. In the present study, material was obtained from the respiratory and lymphatic systems, as this allowed the most efficient sample collection without the need to prolong the immobilization procedure. Extended immobilization may pose a risk for large mammals such as EB due to the possibility of complications such as aspiration pneumonia. 

The present study describes the first successful isolation of MTBC bacteria from living EB. Although MTBC genetic material has previously been isolated from bronchoalveolar lavage (BAL) fluid collected from the lower respiratory tract of *Bison bonasus* [31], procedures to isolate *Mycobacteria* spp. were not reported. While the isolation of MTBC bacteria has been successfully performed in other living wildlife species, the results have indicated rather low sensitivity. Among these studies, MTBC bacteria have been isolated from the tracheal washes of wild meerkats (*Suricata suricatta*) [22], washings from elephant trunks (*Elephas maximus*) [32], BAL from an African lion (*Panthera leo*) [23] and nasal swabs from a giraffe (*Giraffa camelopardalis)* [33]. Although no reports on biopsies from wildlife as part of BTB diagnostic testing were found, this approach was used for TB diagnosis in pets [34].

The TBA samples were collected by nasotracheal intubation. As the method does not require specialized or expensive equipment, it is a more practical approach than endoscopy, especially in the field. It should be noted, however, that aspiration fluid for TBA techniques does not reach all areas of the lungs, which may result in a lack of mycobacteria in the collected material, despite their presence in the airways [35]. The fact that *M. caprae* was successfully isolated from TBA confirms that respiratory shedding is highly probable in EB, thus suggesting the possibility of BTB transmission to other free-ranging species. 

The present study examines the potential value of the biopsy of the lateral retropharyngeal lymph node in EB. *M. caprae* was found to be present in the biopsy material from two out of four infected EB; the first case of biopsy being used in EB for this purpose. In humans, biopsy is sometimes included in the testing for tuberculosis [36], while in animals, it has been performed for various reasons, such as collecting material for detecting mycobacteriosis in birds [37]. 

The lateral retropharyngeal lymph node was selected for biopsy based on the observation that tuberculosis lesions have often been noted in this location in EB previously (Krajewska-Wędzina, unpublished data). Access to these lymph nodes is complicated by the possibility of damage to the anatomical structures in this area, primarily the maxillary vein. The selection of a suitable biopsy needle is also an important consideration, due to the significant thickness of EB skin. Nevertheless, our successful isolation of mycobacteria indicates that the biopsy of the lateral retropharyngeal lymph node described herein is an additional tool for BTB diagnostics in living EB, and one that could offer more value as the collection procedure is refined. 

The direct detection of MTBC DNA (using the ProbeTec PCR) from clinical samples, such as nasal and pharyngeal swabs, TBA, and lymph node biopsy, proved to be a practical testing method. Positive results were obtained from the TBA and pharyngeal swab, suggesting that ProbeTec can be used as a complementary assay. The effectiveness of the ProbeTec test when used in isolation may be lowered due to intermittent mycobacterial shedding (swabs, TBA) [27] or the small amount of genetic material (biopsy).

In other species, studies have been performed using molecular methods to directly detect genetic material in milk and nasal swabs from tuberculous cattle [38], in trunk washings from *M. tuberculosis*-infected Asian elephants [39], and bronchoalveolar lavage and trunk wash from MTBC-infected African elephants [40]. However, in all of those species, such methods are considered to be ancillary tools to other diagnostic techniques. In our studies, we also recommend including culture and molecular methods in the diagnostic algorithm for BTB in living EB, together with other available methods (serology, tuberculin test, gamma-interferon test), as low numbers of tested animals do not allow the determination of sensitivity and specificity of any of those methods in EB. From preliminary studies, it appears that collecting nasal and pharyngeal swabs for a culture and molecular diagnostic is less effective than sourcing material from TBA and biopsy. However, we suggest collecting all three types of samples to increase the possibility of detecting infected animals. In the future, studies on a larger number of animals should be performed as well as collecting additional samples, such as via transbronchial biopsy.

## 5. Conclusions

The techniques described in the present study demonstrate the feasibility of mycobacterial isolation from live European bison infected with *M. caprae,* offering a potentially useful tool for disease surveillance. This is the first report describing the successful isolation of *M. caprae* from infected living EB and the use of biopsy for the isolation of mycobacteria in wildlife. The lateral retropharyngeal lymph nodes and tracheobronchial aspirates appear to be appropriate clinical material. This study demonstrates the added value of combining molecular tests with culture for BTB diagnosis in EB. This will be especially important for use in genetically valuable animals such as EB to exclude false positive results and avoid unnecessary culling. Our findings also represent the first conclusive confirmation of the respiratory shedding of viable *M. caprae* in EB. 

## Figures and Tables

**Table 1:** A comparison of MTBC isolation in samples obtained from different material collected from living and post mortem European bison.

Animal ID	Method	Material				
		Biopsy	TBA	Nasal Swab	Pharyngeal Swab	Lymph Nodes Post Mortem
1	Culture	NEG	NEG	NEG	NEG	NEG
	ProbeTec	NEG	NEG	NEG	NEG	NEG
2	Culture	NEG	NEG	NEG	NEG	NEG
	ProbeTec	NEG	NEG	NEG	NEG	NEG
3	Culture	NEG	NEG	NEG	NEG	NEG
	ProbeTec	NEG	NEG	NEG	NEG	NEG
4	Culture	NEG	NEG	NEG	NEG	POS
	ProbeTec	NEG	POS	NEG	NEG	POS
5	Culture	POS	POS	NEG	NEG	POS
	ProbeTec	NEG	POS	NEG	POS	POS
6	Culture	NEG	POS	NEG	NEG	POS
	ProbeTec	NEG	NEG	NEG	NEG	POS
7	Culture	POS	NEG	NEG	NEG	POS
	ProbeTec	NEG	NEG	NEG	NEG	POS

NEG: NEGATIVE; POS: POSITIVE; TBA: tracheobronchial aspirates; culture: mycobacterial culture; ProbeTec: MTBC PCR.
